# Retraction: Polyethylene glycol-doped BiZn_2_VO_6_ as a high-efficiency solar-light-activated photocatalyst with substantial durability toward photodegradation of organic contaminations

**DOI:** 10.1039/d3ra90015k

**Published:** 2023-03-02

**Authors:** Mahsa Pirhashemi, Sami Elhag, Aziz Habibi-Yangjeh, Galia Pozina, Magnus Willander, Omer Nur

**Affiliations:** a Department of Science and Technology (ITN), Linköping University, Campus Norrköping SE-601 74 Norrköping Sweden mahsa.pirhashemi@liu.se mahsapirhashemi@uma.ac.ir; b Department of Chemistry, Faculty of Science, University of Mohaghegh Ardabili P. O. Box 179 Ardabil Iran; c Department of Physics, Chemistry and Biology (IFM), Linköping University S-581 83 Linköping Sweden

## Abstract

Retraction of ‘Polyethylene glycol-doped BiZn_2_VO_6_ as a high-efficiency solar-light-activated photocatalyst with substantial durability toward photodegradation of organic contaminations’ by Mahsa Pirhashemi *et al.*, *RSC Adv.*, 2018, **8**, 37480–37491, https://doi.org/10.1039/C8RA06896H.

The Royal Society of Chemistry hereby wholly retracts this RSC Advances article due to concerns with the reliability of the data. The Editor has been contacted by Linköping University, Sweden regarding an investigation by the National Board for Assessment of Research Misconduct (NPOF) which has concluded that this RSC Advances article contains fabricated XRD data in Fig 2 and 13b.

The authors do not agree with NPOF’s ruling of research misconduct.

A. Habibi-Yangjeh stated that this research has been carried out in Linköping University, Sweden, under the supervision of Dr Omer Nur, during the sabbatical leave of the first author. In this paper, the contribution of A. Habibi-Yangjeh was discussion about the photocatalysis results and editing of the proof, and he has not contributed in XRD analyses, which are the subject of this retraction.

The other authors have not responded to correspondence regarding this retraction.

Signed: Laura Fisher, Executive Editor, RSC Advances

Date: 16th February 2023

## Supplementary Material

